# Recent Progress on Feasible Strategies for Arbutin Production

**DOI:** 10.3389/fbioe.2022.914280

**Published:** 2022-05-09

**Authors:** Ke-Xin Xu, Meng-Ge Xue, Zhimin Li, Bang-Ce Ye, Bin Zhang

**Affiliations:** ^1^ Jiangxi Engineering Laboratory for the Development and Utilization of Agricultural Microbial Resource, College of Bioscience and Bioengineering, Jiangxi Agricultural University, Nanchang, China; ^2^ College of Bioengineering, East China University of Science and Technology, Shanghai, China

**Keywords:** arbutin, plant extraction, chemical synthesis, biotransformation, microbial fermentation

## Abstract

Arbutin is a hydroquinone glucoside and a natural product present in various plants. Arbutin potently inhibits melanin formation. This property has been exploited in whitening cosmetics and pharmaceuticals. Arbutin production relies mainly on chemical synthesis. The multi-step and complicated process can compromise product purity. With the increasing awareness of sustainable development, the current research direction prioritizes environment-friendly, biobased arbutin production. In this review, current strategies for arbutin production are critically reviewed, with a focus on plant extraction, chemical synthesis, biotransformation, and microbial fermentation. Furthermore, the bottlenecks and perspectives for future direction on arbutin biosynthesis are discussed.

## Introduction

Arbutin is a hydroquinone (HQ) glucoside natural product with two different configurations: alpha (α)- and beta (β)-arbutin (Couteau & Coiffard, 2016). The formation of the two isomers is based on the binding state of HQ and the anomeric carbon atom in the glucose molecule (Hazman et al., 2021). Beneficial properties attributed to the isomers include anti-oxidative (Yu et al., 2015), anti-inflammatory (Lee & Kim, 2012), anti-microbial (Jurica et al., 2017), and anti-cancer (Su et al., 2020) effects. The list of potential applications of arbutin is growing (Saeedi et al., 2021). Particularly, α- and β-arbutin are widely used in pharmaceutical and cosmetic industries due to their potent inhibition of tyrosinase activity (Liu et al., 2016). The biosynthesis of α- and β-arbutin is dependent on the glycosylation of hydroquinone using glycosidases as catalyst and activated sugar as glycosyl donor. Currently, the species and activity of α-glycosidases were significantly higher than that of β-glycosidases, which probably makes α-arbutin more active on tyrosinase than β-arbutin. The tyrosinase inhibition by α-arbutin was 10-times greater than that of β-arbutin, indicating the value of α-arbutin for the cosmetic industry (Funayama et al., 1995).

**FIGURE 1:**
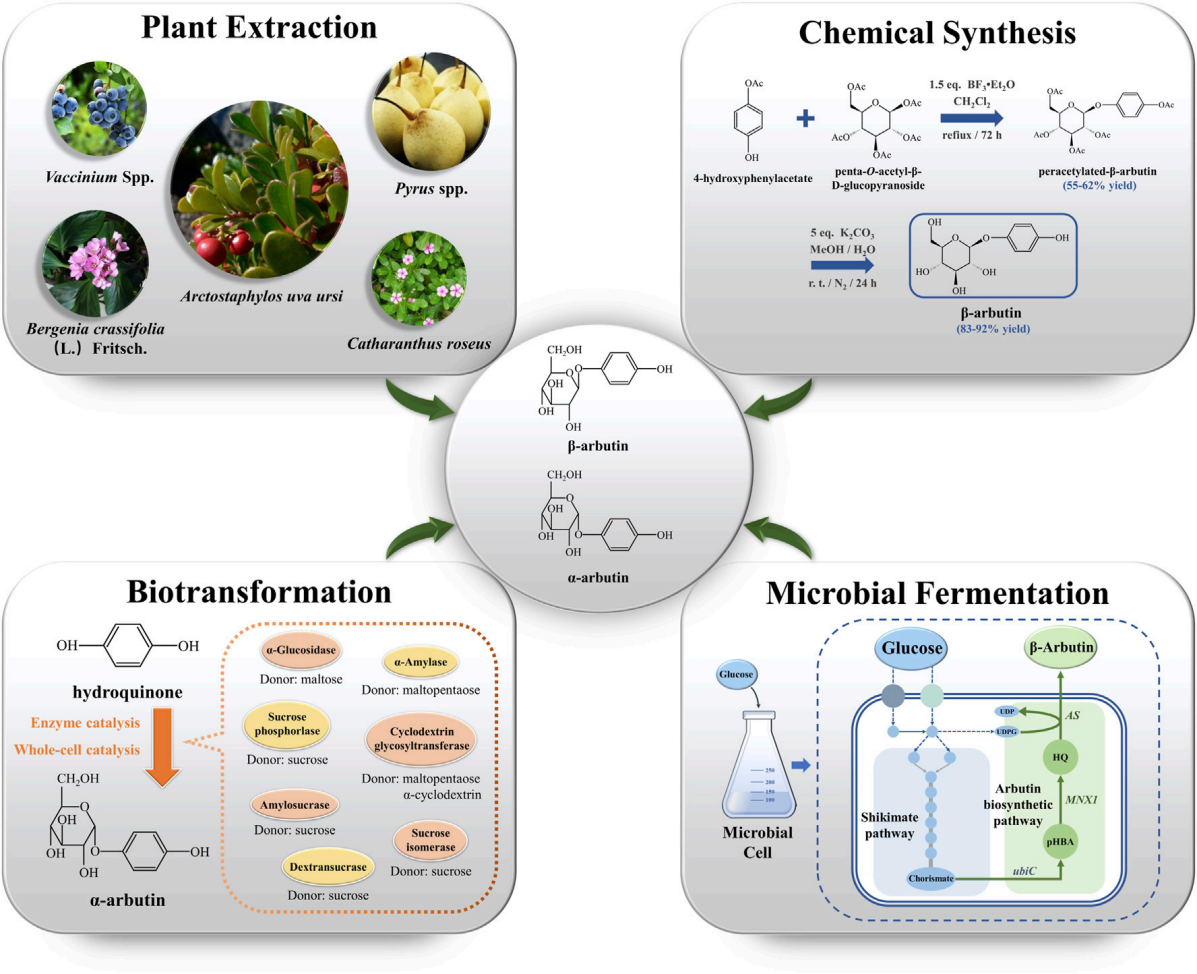
The production of arbutin *via* plant extraction, chemical synthesis, biotransformation, and microbial fermentation. *ubiC* encodes chorismate lyase; *MNX1* encodes 4-hydroxybenzoate 1-hydroxylase; *AS* encodes arbutin synthase. UDPG: uridine diphosphate glucose; UDP: uridine diphosphate; pHBA: p-hydroxybenzoic acid; HQ: hydroquinone.

Increased standards of living have prompted increased demand for cosmetics. This has increased the demand for arbutin. To meet this demand, arbutin production has become the research focus. β-arbutin production mainly depends on plant extraction, chemical synthesis, and microbial fermentation (Figure 1). In contrast, α-arbutin production relies on biotransformation. In this review, we summarize the four synthetic methods of arbutin and systematically analyze their strengths and weaknesses.

## Arbutin Production *via* Plant Extraction

The primary natural source of β-arbutin is bearberry leaves (*Arctostaphylos uvaursi*) (Asensio et al., 2020). It is also found in pears (Cui et al., 2005), wheat, coffee, and tea (Migas & Krauze-Baranowska, 2015) (Figure 1). Although β-arbutin is widely available in plants, its extraction from plants is hindered by its low content, the complicated extraction process, and low purity of the extracted product. To overcome these drawbacks, plant cell culture techniques have been explored. In this approach, the glycosylation ability of plant cells is harnessed to convert exogenous HQ to β-arbutin. HQ catalyzed β-arbutin synthesis in *Catharanthus roseus* cells yielded 9.2 g/L β-arbutin (Inomata et al., 1991). Additionally, hairy roots of *Brugmansia candida* (Casas et al., 1998), *Capsicum annuum* (Lubitz et al., 2016), and *Polygonum multiflorum* have been used as plant cell reactors for β-arbutin synthesis (Table 1). Compared to plant extraction, plant cell culture techniques have the advantage of being independent of climate and other environmental factors in β-arbutin production. However, disadvantages of this method include the long production cycle, low yield, and difficulty in isolation and purification. The HQ addition time must be finally controlled since it has a significant influence on β-arbutin production. Currently, β-arbutin synthesis by plant cell culture method still cannot meet the requirements for industrial-scale production.

**TABLE 1:** Production of arbutin by plant cell reactors, enzymatic synthesis, and whole-cell catalysis.

Source	Enzyme	HQ	Donors	Molar yield	Yield	Reference
hairy roots of *Brugmansia candida*	—	0.36 mM	—	40%	—	Casas et al. (1998)
*Aronia melanocarpa*	—	Total amount of 384 mg/L divided into two portions	—	52.71 ± 11.2%	0.15 g/L	Rytter et al. (2014)
*Capsicum annuum*	—	15.57 mM	—	—	0.37 g/L	Lubitz et al. (2016)
*Catharanthus roseus*	—	1.4 mmol/h added by continuously	—	98%	9.22 g/L	Inomata et al. (1991)
*RauwolJia serpentina*	—	Total amount of 254 mM added by continuously	—	83%	18 g/L	Xu et al. (2013)
*L. mesenteroides*	Sucrose phosphorlase	90 mM	1.46 M sucrose	65%	16 g/L	Kitao & Sekine, (1994)
*T. thermosaccharolyticum*	Sucrose phosphorlase	5 mM	15 mM sucrose	21%	0.29 g/L	Yao et al. (2020)
*L. mesenteroides*	Sucrose phosphorlase	145.5 mM	584.3 mM sucrose	78.3%	31 g/L	Wan et al. (2012a)
*L. mesenteroides*	Sucrose phosphorlase	363.6 mM	1.8 M sucrose	99%	98 g/L	Li et al. (2020a)
*Bacillus subtilis* X-23	α-Amylase	182 mM	121 mM maltopentaose	24.8%	1.5 g/L	Dong et al. (2016)
*Thermoanaerobacter* sp.	Cyclodextrin glycosyltransferase	9 mM	51 mM α-cyclodextrin	21.2%	0.52 g/L	Mathew & Adlercreutz, (2013)
*Thermoanaerobacter* sp.	Cyclodextrin glycosyltransferase	9 mM	278 mM maltodextrin	19.1%	0.47 g/L	Mathew & Adlercreutz, (2013)
*Saccharomyces cerevisiae*	α-Glucosidase	50 mM	1.5 M maltose	4.6%	1 g/L	Prodanović et al. (2005b)
*Saccharomyces cerevisiae*	α-Glucosidase	9 mM	1.5 M maltose	13%	0.4 g/L	Prodanović et al. (2005a)
*L. mesenteroides*	Dextransucrase	450 mM	215 mM sucrose	0.4%	0.5 g/L	Seo et al. (2009)
thermal spring metagenome	Amylosucrase	20 mM	100 mM sucrose	75%	4.1 g/L	Agarwal et al. (2021)
*D. geothermalis*	Amylosucrase	2.3 mM with ascorbic acid	23 mM sucrose	90%	5.8 g/L	Seo et al. (2012)
*Cellulomonas carboniz*	Amylosucrase	5 mM	20 mM sucrose	44.7%	0.61 g/L	Yu et al. (2018)
*Erwinia rhapontici*	Sucrose isomerase	50 mM	1 mM surcose	33.2%	4.52 g/L	Zhou et al. (2011)
*X. campestris* WU-9701	α-Glucosidase	45 mM	1.2 M maltose	55.6%	6.8 g/L	Kurosu et al. (2002)
*X. maltophilia* BT-112	—	120 mM	240 mM sucrose	93.6%	30.6 g/L	Liu et al. (2013b)
*X. maltophilia* BT-112	—	240 mM	480 mM Sucrose	94.5%	61.7 g/L	Liu et al. (2014)
*X. maltophilia* BT-112	—	1.8 M	3.6 M sucrose	93.7%	38.2 g/L	Wei et al. (2016)
*E． coil*	transglucosidase	—	1.1 M glucose	76%	21 g/L	Wu et al. (2008)
*E． coil*	Amylosucrase from *X． campestris*	15 mM	1.2 M of sucrose	95%	83.31 g/L	Zhu et al. (2019a)
*E． coil*	Amylosucrase from *X． campestris*	Total ∼418.2 mM	1.83–2.22 M sucrose	∼95%	102–108 g/L	Zhu et al. (2019b)
*E． coil*	Amylosucrase from *X． campestris*	Total 234 mM	937 mM sucrose	95.5%	60.9 g/L	Yang et al. (2018)
*E． coil*	arbutin synthase	—	30 g/L glucose	—	4.19 g/L	Shen et al. (2017)
*Y． lipolytica*	arbutin synthase	—	100 g/L glucose	—	8.6 ± 0.7 g/L	Shang et al. (2020)
*P． chlororaphis* P3	glucosyltransferase	—	18 g/L glycerol Total 36 g/L glucose	—	6.79 g/L	Wang et al. (2018)

## Arbutin Production *via* Chemical Synthesis

Chemical synthesis is based on glucose with a protecting group and HQ that catalyze glycosylation. This is followed by the removal of the protecting group to obtain arbutin. Glycosylation is the crucial step in arbutin synthesis. The chemical synthesis of β-arbutin was first studied in 1912 (Zhou et al., 2019b). In the absence of solvent, glucose is treated with excess acetyl bromide. Next, the glycosyl bromide is treated with HQ under an alkaline condition to obtain tetra-acetyl arbutin. Finally, treatment with a barium hydroxide solution and acidification by CO_2_ yields β-arbutin.

Subsequent studies on the chemical synthesis of α-arbutin have been reported. Based on the different glycosyl donors used, chemical synthesis can be divided into the Koenigs-Knorr glycosylation, which uses bromo glucoside as the glycosyl donor, and the Helferich method, using an acyl group. The Helferich method dominates arbutin production due to its simplicity, low cost, and high product purity (Qiao et al., 2013). Tetra-*O*-benzyl-a-D-glucopyranosyl trichloroacetimidate as glucosyl donor with HQ reportedly obtained α-arbutin with a yield of 65% in a two-step reaction catalyzed by trimethylsilyl trifluoromethanesulfonate (Wang et al., 2006). The use of penta-*O*-acetyl-β-D-glucopyranoside and 4-hydroxyphenylacetate as aglycone donors for β-arbutin synthesis has also been studied (Figure 1). The substitution of penta-*O*-acetyl-α-D-glucopyranoside for penta-*O*-acetyl-β-D-glucopyranoside resulted in the production of unnatural α-arbutin without the production of its isomers (Chen et al., 2015). The use of 4-hydroxyphenylacetate is hindered by the lack of commercial availability; it must be synthesized through additional chemical steps. Although chemical synthesis can overcome the many disadvantages of natural extraction, it has a few drawbacks, such as the pronounced HQ toxicity, the labor-intensive nature of the process, strict reaction conditions, and low isomer selectivity. Thus, research has tended to focus on exploring arbutin production methods that are efficient, simple, and environment-friendly.

## Arbutin Production *via* Biotransformation

Biotransformation is an environment-friendly and mild synthesis approach that utilizes enzymes or whole cells as catalysts to convert HQ to arbutin. Compared with the chemical synthesis approach, arbutin production *via* biotransformation has the advantages of low energy consumption, reduced pollution, and high specificity.

### Enzyme Catalysis

Enzyme catalysis is the main route to synthesize α-arbutin. Glycosyltransferases (GTs) are used to produce α-arbutin from HQ and diverse glycosyl donors. GTs catalyze the formation of glycosidic bonds between glycosyl donors and acceptors. GTs are classified into two types, Leloir and non-Leloir, based on the catalytic properties (Xu et al., 2016). The Leloir type of GTs is most common. These use activated donor substrates, such as uridine diphosphate glucose, uridine diphosphate galactose, and uridine diphosphate rhamnose, for α-arbutin biosynthesis (Bungaruang et al., 2013). Non-Leloir type GTs do not require co-factors or activated substrates. Instead, they directly utilize the free energy from the cleavage of glycosyl donors, such as sucrose, starch, and their hydrolysis products, for α-arbutin biosynthesis (Plou et al., 2002). Currently, various GTs, including sucrose phosphorylase, α-amylase, cyclodextrin glycosyltransferase, α-glucosidase, dextransucrase, amylosesucrase, and sucrose isomerase, derived from different sources are used for α-arbutin production (Zhu et al., 2018) (Table 1) (Figure 1).

Sucrose phosphorylase (SPase), primarily derived from *Leuconostoc* sp., was the first GT demonstrated to be capable of transferring phosphorylated glucose or glucose groups produced by sucrose decomposition to other receptors to produce α-arbutin (Sugimoto et al., 2008). In 1994, the SPase of *Leuconostoc mesenteroides* was first demonstrated to be capable of catalyzing α-arbutin biosynthesis (Kitao & Sekine, 1994). The complex metabolic regulation mechanism of wild-type *L. mesenteroides* strains leads to limited SPase activity and low yields of α-arbutin. To improve the enzymatic activity and stability of SPase, a molecular biological technology approach was applied to construct a robust heterogeneous host to express SPase (Wan et al., 2012b). In this approach, SPase was overexpressed in *Escherichia coli* BL21 (DE3), and 98 g/L α-arbutin was synthesized by the purified recombinant enzyme under optimal conditions (Li et al., 2020b). In addition, co-expression of SPase from *Thermoanaerobacterium thermosaccharolyticum* (TtSPase) and molecular chaperone pG-TF2 (GroES-GroEL-Tig) resulted in 21% molar conversion of α-arbutin (Yao et al., 2020).

Cyclodextrin glucosyltransferase (CGTase), primarily derived from *Thermoanaerobacter* sp., is an extracellular enzyme that catalyzes the breakdown of starch and linear maltodextrin to produce cyclodextrins. CGTase catalyzes the transglycosylation of carbohydrates and non-carbohydrate compounds using its disproportionation activity. CGTase from *Thermoanaerobacter* sp. catalyzes transglycosylation of HQ and maltodextrin or α-cyclodextrin to produce α-arbutin. When α-cyclodextrin was used as a donor, the molar yield of α-arbutin was 21.2%. In addition, the use of a two-step enzymatic reaction system consisting of CGTase and amyloglucosidase increased the molar yield of α-arbutin to 30% (Mathew & Adlercreutz, 2013).

Amylosucrase (ASase), primarily derived from *Deinococcus* sp., is a transglucosidase that uses sucrose as the only glycosyl donor. ASase has superior catalytic efficiency for arbutin production (Tian et al., 2018). For instance, a novel ASase (AS_met_) identified from the microflora metagenome of a thermal aquatic habitat displayed a 70% conversion efficiency from HQ to α-arbutin (Agarwal et al., 2019; Agarwal et al., 2021). Investigation of an ASase from *Deinococcus geothermalis* described that HQ in the reaction mixture was unstable, and the production yield was only 1.3%. However, the yield reached 90% with the addition of 0.2 mM ascorbic acid and a 10:1 M ratio of sucrose and HQ molecules (Seo et al., 2012). This study demonstrated that the oxidation of HQ was the main barrier in the catalytic reaction and that the addition of antioxidants could effectively inhibit oxidation and improve the yield of α-arbutin.

The greatest advantage of enzyme catalysis is that it can exclusively synthesize α-arbutin using reaction conditions that are milder and faster than chemical synthesis. However, the yield of α-arbutin varies with the type or source of enzyme, glycosyl donor, reaction time, and reaction temperature. Simultaneously, the toxic of HQ and high cost of activated substrates such as UDP-containing glycosyl donor restricted the application of enzyme catalysis. To address these problems, it is feasible to adopt flow addition strategy to control the concentration of HQ and develop whole cell catalysis for the synthesis of active glycosylated donors using endogenous metabolic pathways.

### Whole-cell Catalysis

Microbial cells can also be directly used as catalysts. Cell lysis and enzyme purification are not required. Furthermore, the process can easily be scaled-up to produce valuable products (Wachtmeister & Rother, 2016). Recently, wild-type non-model and engineered model microorganisms have been used as cellular catalysts for the bioconversion of α-arbutin. For instance, lyophilized cells of *Xanthomonas campestris* WU-9701 harboring α-glycosidase have been utilized as biocatalysts to produce 42 mM α-arbutin from HQ in the presence of 1.2 mM maltose (Kurosu et al., 2002; Sato et al., 2012). Notably, mutant *Xanthomonas maltophilia* BT-112 produced α-arbutin at a titer of 61.7 g/L during dissolved oxygen-control pulse fed-batch fermentation. This result represents the highest reported α-arbutin titer using wild-type non-model microorganisms (Liu C.-Q. et al., 2013; Liu et al., 2014; Wei et al., 2016; Zhou et al., 2019a). The essence of whole-cell catalytic α-arbutin synthesis is using enzyme-catalyzed reactions in microorganisms. Although α-arbutin can be synthesized by whole-cell catalysis of wild-type strains, there are still some disadvantages that restrict its application include low GT expression and the unknown genetic background of the strains. To address this problem, recombinant *E. coil* harboring heterogenous GTs were employed as whole-cell catalysts to produce α-arbutin (Wu et al., 2008; Yang et al., 2018). Interestingly, batch-feeding whole-cell catalytic synthesis of α-arbutin using recombinant *E. coli* expressing ASase from *X. campestris* pv. 8004 (Amy-1) generated 306 mM of α-arbutin in 15 h with a 95% conversion rate of HQ (Zhu et al., 2019a). This production process was scaled-up in a 5000 L reactor and achieved 108 g/L of α-arbutin with a 95% molar conversion rate (Zhu et al., 2019b).

These results highlight the remarkable progress in generating and utilizing recombinant microorganisms harboring highly-expressed GTs for the whole-cell catalytic synthesis of α-arbutin. However, α-arbutin synthesis using whole-cell catalysis has certain limitations. The biosynthesis pathway of uridine diphosphate glucose (UDPG) in microorganisms are uncontrollable, and GT introduction frequently leads to unsatisfactory results. Modifying the biosynthesis pathway of UDPG to enhance GT catalytic efficiency is an active area of research.

### Improving α-Arbutin Production *via* Immobilized Substrates, Enzymes, and Cells

α-arbutin production *via* biocatalysis has the advantages of single product composition and easy product separation. Nevertheless, excess HQ can lead to apoptosis of cells, limiting the substrate conversion efficiency, and affecting α-arbutin synthesis. To address this problem, a slow-release system was constructed by immobilizing a substrate capable of inhibiting cellular and enzymatic activities on a carrier that effectively reduces toxic substrate concentrations. Immobilizing HQ to the H107 resin reportedly improved the maximum tolerated concentration to 254 mM HQ in *X. maltophilia* BT-112. The α-arbutin productivity was 526% higher than that obtained using free HQ (Liu C. et al., 2013). In addition, immobilizing HQ to chitosan-coated magnetic nanoparticles allowed the production of 7.85 g/L α-arbutin during the catalysis of *X. maltophilia* BT-112 cells (He et al., 2021). In addition to immobilized substrates, immobilization approaches also have been developed to improve the stability and efficiency of biocatalysts for α-arbutin production. The ASase from *Deinococcus geothermalis* DSM 11300 (DgAS) could catalyze 400 mM HQ to produce 88.6 g/L α-arbutin, with 81% of 400 mM HQ converted in 30 min (Lee et al., 2018). Immobilization of DgAS on Amicogen LKZ118 beads resulted in the production of 95 g/L α-arbutin from 400 mM HQ, with the yield maintained between 85 and 90% in a single cycle (Lee et al., 2018).

## Arbutin Production *via* Microbial Fermentation

Although high yields of α-arbutin have been obtained using biotransformation, the addition of HQ and extraction of GTs increases the cost of the process. Excess HQ added to improve the yield of arbutin can be toxic to cells, thereby inhibiting the catalytic activity of cells or enzymes. To address these problems, engineering high-performance arbutin-producing strains that produce arbutin via microbial fermentation is being explored as a promising approach (Figure 1).

### Reconstructing the Anabolic Arbutin Pathway

To construct arbutin-producing microorganisms, the synthetic arbutin pathway must be elucidated. In 1997, a flavin adenine dinucleotide (FAD)-dependent 4-hydroxybenzoate 1-hydroxylase encoded by *MNX1* was identified in *Candida parapsilosis* CBS604 (Figure 1). The enzyme catalyzes HQ synthesis from p-hydroxybenzoic acid (pHBA) (Eppink et al., 1997). In addition, arbutin synthase encoded by *AS* is a novel member of Class IV GTs belonging to the nucleotide recognition domain type 1β family (NRD1β) (Eberhardt et al., 2014) was discovered in *Rauvolfa serpentina* (Figure 1). This enzyme was demonstrated to catalyze β-arbutin synthesis from HQ using uridine diphosphate glucose as the glycosyl donor (Arend et al., 2001). The discovery of these two enzymes has made it possible to synthesize arbutin from glucose. *MNX1* and *AS* were introduced into *E. coli*; the resulting bacteria were capable of synthesizing 54.71 mg/L of β-arbutin from glucose (Shen et al., 2017). This anabolic pathway was also shown to be feasible in *Yarrowia lipolytica*. Since the chorismate pyruvate lyase (UbiC), which can catalyze pHBA from chorismate, is unavailable, *UbiC* from *E. coli* should be introduced along with *MNX1* and *AS* in *Y. lipolytica*. β-arbutin titer produced by engineered *Y. lipolytica* was 72.3 ± 2.1 mg/L after 72 h of fermentation (Shang et al., 2020). Additionally, a plasmid- and inducer-independent β-arbutin synthesis pathway involving *MNX1*, *AS*, and *XanB2* (encoding chorismate pyruvate lyase), was constructed in *P. chlororaphis* P3. This pathway produced β-arbutin at a titer of 130 mg/L (Wang et al., 2018). These collective findings suggest that arbutin synthesis pathways from plants can be assembled into microorganisms.

### Modifying the Shikimate Pathway to Enhance Shikimate Production

Due to the insufficient supplementation of precursors, only a low yield of β-arbutin results from the assimilation of the arbutin synthesis pathway. Metabolic engineering strategies have been widely used to construct high-performance microbial strains to produce natural and non-natural compounds (Huccetogullari et al., 2019). Chorismate, the precursor in the arbutin biosynthetic pathway, is synthesized by the shikimate metabolic pathway. Modifying this pathway by metabolic engineering has been demonstrated as an effective approach to increase arbutin production. To improve β-arbutin production, overexpression of *aroL* (which encodes shikimate kinase II), *ppsA* (which encodes phosphoenolpyruvate synthetase), and *tktA* (which encodes transketolase) genes in the upstream pathway, and feedback inhibition resistant *aroG* ^*fbr*^ (which encodes 2-dehydro-3-deoxyphosphoheptonate aldolase) in *E. coli* SXL92 increased β-arbutin yield by 60-fold, with production reaching 3.29 g/L (Shen et al., 2017). Similarly, overexpression of *DHS1* and *DHS2* (which encode 3-deoxy-D-arabino-heptulosonic acid 7-phosphate synthase) in engineered *Y. lipolytica* increased β-arbutin yield to 819.6 mg/L (Shang et al., 2020). The collective results demonstrate that modifying the upstream pathway is beneficial in increasing β-arbutin production.

### Optimization of Glucose Addition to Promote β-Arbutin Biosynthesis

Since glucose is both a carbon source for cell growth and a substrate for β-arbutin production, optimizing the glucose supply is critical in improving β-arbutin production. Providing 30 g/L glucose was optimal for β-arbutin production by engineered *E. coli*; β-arbutin production reached 4.19 g/L in 48 h (Shen et al., 2017). Similarly, 100 g/L glucose proved to be optimal for engineered *Y. lipolytica*, with β-arbutin production reaching 8.6 ± 0.7 g/L, the highest yield reported to date (Shang et al., 2020). In addition, a mixture of glucose and pHBA supplemented by batch fermentation increased β-arbutin production in engineered *P. chlororaphis* P3 to 6.79 g/L (Wang et al., 2018).

## Conclusion and Future Prospects

Industrial arbutin production relies on chemical synthesis, which is hindered by the complicated operation steps, poor product stereoselectivity, and environmental concerns. In contrast, arbutin production through biotransformation has the advantages of easy access to raw materials, moderate fermentation conditions, short production period, high catalytic capacity, and environmental friendliness. Arbutin obtained by biotransformation is also easily purified as a single structure, which is crucial for industrial applications. The most effective and industrial-scale α-arbutin production has been achieved in recombinant *E. coli* expressing ASase from *X. campestris*. However, arbutin synthesis *via* biotransformation requires excellent biocatalysts, including high-performance GTs and recombinant microbial cells. These excellent biocatalysts could be obtained through directed evolution or protein structure-based targeted mutation of existing GTs. Furthermore, high-performing whole-cell catalysts can be achieved by optimizing GT expression, increasing the metabolic flux of the UDPG biosynthesis pathway, and co-expressing molecular chaperones.

Microbial fermentation provides a new candidate for direct arbutin synthesis from renewable carbon sources. This approach does not require additional HQ supplementation and enzyme extraction steps. The arbutin biosynthetic pathway has been assembled in various microorganisms, including *E. coil*, *Y. lipolytica,* and *P. chlororaphis*. However, the β-arbutin production titer is typically low. There are bottlenecks, such as tolerance, precursor supplement, and biosecurity, in the biosynthesis of aromatic compounds in *E. coli* and *Y. lipolytica*. To promote the microbial fermentation of arbutin, better microbial platforms must be developed. In recent years, inspired by the application of synthetic biology tools, a multitude of “generally regarded as safe” strains, such as *Corynebacterium glutamicum* and *Bacillus subtilis*, have been engineered to utilize the shikimate pathway to produce high value-added products. For example, recombinant *C. glutamicum* can produce 141 g/L shikimate (Kogure et al., 2016) and 82.7 g/L protocatechuic acid (Kogure et al., 2021). These yields are significantly higher than the yields obtained by other microorganisms. The biosynthesis of arbutin is derived from the shikimate pathway, like shikimate and protocatechuic acid. Thus, *C. glutamicum* has superior potential for arbutin biosynthesis.

## Abbreviations

(HQ), hydroquinone ; (GTs), Glycosyltransferases ; (SPase), Sucrose phosphorylase ; (CGTase), Cyclodextrin glucosyltransferase ; (ASase), Amylosucrase ; (FAD), adenine dinucleotide ; (NRD1β), nucleotide recognition domain type 1β family ; (pHBA), p-hydroxybenzoic acid ; (UDPG), uridine diphosphate glucose.
